# Construction and Characterization of a Population-Based Cohort to Study the Association of Anesthesia Exposure with Neurodevelopmental Outcomes

**DOI:** 10.1371/journal.pone.0155288

**Published:** 2016-05-11

**Authors:** Danqing Hu, Randall P. Flick, Stephen J. Gleich, Maura M. Scanlon, Michael J. Zaccariello, Robert C. Colligan, Slavica K. Katusic, Darrell R. Schroeder, Andrew C. Hanson, Shonie L. Buenvenida, Robert T. Wilder, Juraj Sprung, David O. Warner

**Affiliations:** 1 Department of Anesthesiology, Mayo Clinic, Rochester, Minnesota, United States of America; 2 Mayo Graduate School, Mayo Clinic, Rochester, Minnesota, United States of America; 3 Department of Psychology, Mayo Clinic, Rochester, Minnesota, United States of America; 4 Department of Health Sciences Research, Mayo Clinic, Rochester, Minnesota, United States of America; Massachusetts General Hospital, UNITED STATES

## Abstract

Exposure to general anesthesia at an early age has been associated with adverse neurodevelopmental outcomes in both animal and human studies, but some of these studies employed anesthetic agents that are no longer in clinical use. In this manuscript, we describe the methods used to construct a new population-based study cohort to study the association between early anesthetic exposure and subsequent neurodevelopmental outcomes. A birth cohort of all children born in Olmsted County, MN from January 1, 1996 to December 31, 2000 was identified. For each, school enrollment status in the Independent School District (ISD) 535 at age 5 or 6 and all episodes of anesthetic exposure before age 3 were identified. A study cohort was created by matching children enrolled in ISD 535 based on the propensity of receiving general anesthesia. Three analyses were performed to characterize the study cohort by comparing the birth and parental information, comorbidities, and socioeconomic status. The first analysis compared the characteristics of birth cohort children who were and were not enrolled in ISD 535. The second analysis evaluated the success of the propensity matching schemes in creating groups of children that were similar in measured characteristics except for anesthesia exposure. The third analysis compared the characteristics of children with anesthesia exposures who were and were not included in the final cohort based on propensity matching. Results of these analyses demonstrate only slight differences among the comparison groups, and therefore these are unlikely to compromise our future analysis of anesthetic exposure and neurodevelopmental outcomes.

## Introduction

Most anesthetics produce apoptosis and other neurodegenerative changes when administered to young animals [1–4]. These histological findings have been described in several species, including nonhuman primates, and can be associated with deficits in learning, memory, attention, and other aspects of behavior [5–7] in both rodents and primates. The potential clinical significance of these observations is a topic of intense debate and concern [8–14]. In a population-based birth cohort of children born from 1976 to 1982, our group found an association between multiple, but not single, exposures to anesthesia prior to age 3 and subsequent learning disabilities (LD) [8, 15] and attention deficit-hyperactivity disorder (ADHD) [9]. Multiple exposures were associated with an approximately 2-fold increase in the incidence of both LD and ADHD. These results are consistent with animal studies showing cognitive and behavioral impairment associated with multiple anesthetics [6, 16]. However, there were limitations inherent in this series of studies. In particular, the primary anesthetic agent for these children was halothane, which is no longer in clinical use. Moreover, some modern monitoring devices (e.g. pulse oximetry, capnography) were not available that time. Thus, it is not clear how these results may relate to contemporary anesthetic practice. In addition, the number of children exposed to anesthesia was insufficient to determine if anesthetic exposure is associated with a particular type of learning disabilities (reading, mathematical, or written language). Finally, given the well-acknowledged limitations of such observational studies [8, 15], especially the potential for confounds, it is critically important to repeat these fundamental observations, which have proved to be an important driver of research in this field.

This manuscript describes the methods used to assemble a new population-based study cohort of children that will be used to determine the association between exposure to general anesthesia as delivered in contemporary practice prior to age 3 and learning/behavioral outcomes including LD, ADHD, and group-administered achievement tests.

## Methods and Materials

### Hypotheses

The planned study will test the following hypothesis: Multiple, but not single, exposures to general anesthesia prior to age 3 years (i.e., prior to the child’s third birthday) are associated with adverse neurodevelopmental outcomes, including LD, ADHD, need for Individualized Education Programs (IEPs), and impaired performance in the group-administered achievement tests.

In constructing the study cohort to evaluate this hypothesis, three additional hypotheses were generated and tested related to equivalence between various groups: 1) children who were born in Olmsted County, MN, and were and were not enrolled in the local school district do not differ in birth and parental characteristics; 2) children in a study cohort who were multiply-, singly-, or unexposed to general anesthesia do not differ in birth and parental information, comorbidities, or socioeconomic status, and 3) exposed children who could not be propensity matched to unexposed children do not differ in birth and parental information, comorbidities, or socioeconomic status from exposed children who could be propensity matched.

### Study cohort creation

#### Initial identification of a study cohort

The Mayo Clinic and Olmsted Medical Center institutional review boards approved this study. Written informed consent was given by participants or caregivers in the case of children for their clinical records to be used in this study.

A prior publication described the methods used to create a birth cohort of children born from January 1, 1994 to December 31, 2007 in Olmsted County, MN for the Mayo Anesthesia Safety in Kids (MASK) study [17]. The primary aim of the MASK study is to sample 1,000 children from this birth cohort for detailed prospective neurodevelopmental assessments of behavior and learning to determine associations between anesthesia exposure and these assessments. In this work, the resources of the Rochester Epidemiology Project (REP) [18] and birth certificate information were utilized to identify all children born in Olmsted County, MN over the period of interest. Available birth certificate information included the child’s name, sex, date of birth, the child’s both parent names, and the mother’s address at the time of the child’s birth.

The current manuscript describes how a study cohort was constructed as a subset of the 1994–2007 birth cohort to support the planned study. Although children in both the planned study and the MASK study are drawn from the same birth cohort, they are not necessarily the same children, and the MASK study does not assess the same learning and behavioral outcomes. In particular, the MASK study involves a four-hour detailed neuropsychological assessment to measure multiple cognitive domains. In contrast, the study cohort described in the current paper will be used to examine learning and behavioral outcomes ascertained through school record review, including LD, ADHD, and group-administered achievement tests.

First, children in the birth cohort who were born from January 1, 1996 to December 31, 2000 were identified. This time range was chosen to allow sufficient duration of follow up to identify the outcomes of interest, and to coincide with widespread clinical replacement of the volatile anesthetic halothane with sevoflurane in pediatric practice. The following children were then excluded: 1) children who moved from Olmsted County prior to their third birthday (to ensure that complete information regarding potential exposure to anesthesia and surgery at a young age was available), 2) children who died prior to their 5th birthday, and; 3) children not enrolled in the local school district at age 5 or 6 (from 5th birthday to the day before 7th birthday), as their records would not be available to ascertain outcomes of interest. The following section describes how enrollment status was determined.

#### Ascertainment of school enrollment status

A contractual research agreement between the Mayo Clinic and the Independent School District (ISD) 535, which provides public schooling for most children in Olmsted County, MN. This agreement makes accessible for review the cumulative school records of all students living within the boundaries of the school district including those attending any of the parochial or private schools, or were home-schooled within the boundaries of ISD 535. The district provides educational services for all children living within its boundaries evaluated for or requiring any specialized services regardless of which school they attend. For purposes of this paper, these children will be considered to be “enrolled” in ISD 535 (regardless of whether they actually attend ISD 535, a private school or are home schooled). Records are maintained if students move from the school district, graduate, or die. The record includes enrollment records, school assessments, individual and group IQ/achievement test results, special educational intervention, reported long-term outcomes (e.g., drop out, expulsion, high school graduation status, etc.), data and notes related to any type of difficulty in learning, performance, or behavior, as observed by teachers, parents, school psychologists, school nurses, etc., medical records relevant to development, learning or behavior, and correspondence with private tutorial and intervention facilities.

To determine the school enrollment status of birth cohort members, identifiers (including birth certificate information) were merged with the ISD 535 school record files. The merging process involved both automated and manual efforts. The automated process utilized an algorithm developed by the Rochester Epidemiology Project that takes into account “fuzzy” measures of proximity and relative frequency of certain variables such as the last name, first name, date of birth, mother’s name, and address [19]. Cohort children not merged with a school file were manually verified to adjust for misspellings, name and address changes, or other errors. Those children not enrolled in ISD 535 at age 5 or 6 were not eligible for inclusion in the study cohort.

#### Ascertainment of anesthesia exposures

The medical records of all children in the birth cohort were reviewed to determine their exposure to general anesthesia prior to the age of 3 years using the Mayo Clinic Surgical Information Retrieval System and a similar resource for procedures performed at Olmsted Medical Center, the only other medical facility within Olmsted County. Details of anesthetic management, including duration, surgical procedure, and drugs utilized, were abstracted.

#### Propensity matching and the study cohort

The potential for confounds is inherent in observational studies. In the planned study, the main concern is that exposure to anesthesia and surgery is simply a marker for other factors that primarily contribute to adverse outcomes. To mitigate these potential effects, a propensity matching strategy was used to select children who met other eligibility criteria for inclusion in the study cohort. A similar strategy has been described for selection of children to be invited to participate in the MASK study [17]. Using all members of the birth cohort who were born between January 1, 1996 to December 31, 2000 and who did not meet any of the previously described exclusion criteria, multinomial logistic regression models were used to calculate propensity for receiving general anesthesia. For these models, the dependent variable had 3 levels indicating the number of anesthetic exposures under the age of 3 (none, single, multiple). The explanatory variables included in the model consisted of two sets: 1) 32 binary indicator variables representing the Johns Hopkins ACG Case-Mix System co-morbidity clusters (aggregated diagnostic groups, ADGs) to quantify the overall health status of each cohort child (as described in other prior work [8]); and 2) birth certificate variables including gestational age, birth weight, Apgar scores at 1 and 5 minutes, complications of pregnancy, age of parents, and level of parental education. Because sex has been reported as an important factor associated with LD and ADHD, and we anticipated that the magnitude of confounding for some variables may differ between sexes, separate propensity models were fit for each sex. Since the dependent variable had 3 levels, two propensity scores were calculated: a propensity score for receiving a single exposure and a propensity score for receiving multiple exposures. In order to create sex-specific strata which take into account both propensity scores the following approach was used. For each propensity score, a 5-level nominal variable was created which categorized subjects according to the quintiles of the observed distribution of propensity score. By using all possible combinations of these 2 categorical variables 25 propensity-matched strata were identified for each sex, with the notation (q_single_, q_multiple_) used to denote these strata. For example, the strata F(1,1) corresponds to females whose propensity score for a single exposure to anesthesia was in the lowest quintile and whose propensity score for multiple exposures to anesthesia was also in the lowest quintile. Similarly, the strata M(1,5) corresponds to males whose propensity score for a single exposure to anesthesia was in the lowest quintile and whose propensity score for multiple exposures to anesthesia was in the highest quintile.

Previous publications suggested an association between multiple exposures to general anesthesia and neurodevelopmental outcomes like LD and ADHD [8, 9, 15]. Therefore the matching strategy aimed to maximize the number of children exposed to general anesthesia in the study cohort, especially those who had multiple anesthetic exposures. Within each of the 50 sex-specific propensity-matched strata, the distribution of children in each exposure (none, single, multiple) group were identified. In strata with at least one child in each of the three exposure groups, all of the exposed individuals (both single and multiple) were selected into the final study cohort along with a random sample of an equal number of children who were not exposed. Using this approach, the goal was to include an equal number of exposed and unexposed children within each stratum. In some strata the number of unexposed children was less than or equal to that of the exposed; for these strata, all available unexposed children were selected into the study cohort. If a given stratum did not contain at least one child from all three exposure groups of children, this stratum was excluded from the study to ensure maximum validity of propensity matching. This was chosen as a conservative approach, but as a result some of the children with anesthesia exposures were not included in the final propensity-matched study cohort.

After the propensity matching had been performed, a measure of socioeconomic status (SES) was calculated for children included in the study cohort and compared among exposure categories. The HOUSES index, developed and validated within the Olmsted County population [20], is calculated by linking address information of the children residence (at age 5 or the age closest to this value) to property data publicly available at local government assessors’ office.

### Statistical analyses

#### Sample size/power considerations

To assess whether general anesthesia prior to age 3 years is associated with neurodevelopmental outcomes in the planned study we will compare four outcomes (cumulative frequency of LD and ADHD, number of recipients of an IEP, and scores of group administered achievement tests) between the two groups of children exposed to anesthesia (single or multiple exposures) and those children not exposed to general anesthesia. In a prior study done in a similar birth cohort born from 1976 to 1982, the cumulative frequency of LD prior to age 19 was 36.6%, 23.6%, and 21.3% for children in the multiple, single, and none exposure group, respectively [8]. Assuming that the cumulative frequency of LD is similar, approximately 250 cases of LD would be expected in the current study cohort. With this expected number of cases, the sample size of the present study will provide statistical power of >80% to detect a hazard ratio as low as 1.6 between children with multiple exposures and unexposed children. Similarly, the current study will provide statistical power of >80% to detect a hazard ratio of 1.5 or larger between children with a single exposure compared to those unexposed. For group-administered achievement tests, this sample size will also provide statistical power of 80% to detect a difference of 0.3 standard deviation units between multiply exposed children and children not exposed.

#### Analysis

Future reports of the planned study will present the methods used to define and analyze each neurodevelopmental outcome. The present manuscript presents the results of three analyses that characterize the study cohort itself.

The first analysis compared the characteristics of children in the birth cohort who were and were not enrolled in ISD 535 at the age of 5 or 6 years to examine potential bias due to this inclusion requirement for the planned study. Potential reasons that children were not enrolled include death prior to age 5, living or moving out of the boundaries of the school district (small portions of Olmsted County are included in other public school districts), or electing to attend another public school outside of ISD 535 (as allowed by MN state law). Characteristics of children who were and were not enrolled in ISD 535, including birth certificate information, were compared using standard statistical methods. The chi-square test was used for categorical variables and the Wilcoxon rank-sum test was used for ordinal and continuous variables.

The second analysis evaluated the success of the propensity matching schemes in creating groups of children that were similar in measured characteristics (except for anesthesia exposure). In order to take into account the stratified study design, characteristics of the three exposure groups (multiple, single, or none) were compared using Cochran-Mantel-Haenszel tests and stratified generalized linear models were used for categorical and continuous variables respectively.

Finally, since some propensity-matched strata did not contain at least one child from each exposure group, there were some multiply and singly exposed children who were not included in the study cohort. To assess potential biases resulting from this exclusion, the characteristics of children with anesthesia exposures (both single and multiple) who were and were not included in the propensity-matched analysis were compared using the chi-square test for categorical variables and the Wilcoxon rank-sum test for ordinal and continuous variables.

## Results

### Comparison of birth cohort children who were and were not enrolled in ISD 535

There were a total of 6,789 children born in Olmsted County, MN from January 1, 1996 to December 31, 2000 who resided in Olmsted County up to their third birthday and provided research authorization for use of their medical records. Of these children, 315 were excluded because their enrollment status in ISD 535 was not known at the time of study cohort selection. Of the remaining children, 4,142 (64%) enrolled in ISD 535 during their fifth and sixth years (from 5th birthday to the day before 7th birthday). Table 1 presents parental characteristics and pregnancy and delivery information in children who were and were not enrolled in ISD 535. Compared to enrollees of ISD 535, the parents of non-enrollees were slightly younger, and both parents had attained higher levels of education. In addition, a higher percentage of children enrolled in ISD 535 were exposed to general anesthesia at least once, as compared to children not enrolled in ISD 535. There were no differences between the enrollees and non-enrollees in estimated gestational age, birth weight, Apgar scores at 1 and 5 minute, or labor/delivery complications.

**Table 1:** Characteristics of children in 1996–2000 birth cohort who were and were not enrolled in ISD 535 (n = 6474).*

	Not enrolled (N = 2332)	Enrolled (N = 4142)	*P* Value†
**General parental characteristics**			
Mother’s age (y)	29(26, 32)	30(26, 33)	<.001
Father’s age (y; n = 6160)	31(28, 34)	32(28, 35)	<.001
Mother's education (y)			0.015
<12	153(7%)	360(9%)	
12	456(20%)	757(18%)	
13–15	555(24%)	1007(24%)	
16	603(26%)	1153(28%)	
>16	509(22%)	754(18%)	
Unknown	56(2%)	111(3%)	
Father's education (y; n = 6189)			<.001
<12	87(4%)	190(5%)	
12	472(21%)	816(21%)	
13–15	476(21%)	830(21%)	
16	394(18%)	1038(26%)	
>16	717(32%)	880(22%)	
Unknown	82(4%)	207(5%)	
Hispanic origin of mother			0.53
No	2267(97%)	4013(97%)	
Yes	64(3%)	125(3%)	
Not specified	1(0%)	4(0%)	
Hispanic origin of father (n = 6189)			0.82
No	2134(96%)	3783(96%)	
Yes	57(3%)	105(3%)	
Not specified	37(2%)	73(2%)	
Race of mother, white			0.19
No	280(12%)	452(11%)	
Yes	2040(87%)	3665(88%)	
Not specified	12(1%)	25(1%)	
Race of father, white (n = 6189)			0.90
No	243(11%)	436(11%)	
Yes	1938(87%)	3439(87%)	
Not specified	47(2%)	86(2%)	
Number of Prenatal Care Visits (n = 6176)	11(9, 13)	11(10, 13)	0.06
Number previously born (n = 6429)			0.79
None	915(39%)	1609(39%)	
1	788(34%)	1375(33%)	
2	355(15%)	716(17%)	
3 or more	259(11%)	412(10%)	
Marital status (n = 6473)			0.37
Married	1955(84%)	3436(83%)	
Not married	377(16%)	705(17%)	
Any medical risk factor	474(20%)	832(20%)	0.82
**Pregnancy and delivery information**			
Sex			0.08
Male	1221(52%)	2075(50%)	
Female	1111(48%)	2067(50%)	
Estimated gestational age (w; n = 6429)	39(38, 40)	39(38, 40)	0.98
Apgar, 1 minute (n = 6449)	8(7, 9)	8(8, 9)	0.87
Apgar, 5 minutes (n = 6443)	9(9, 10)	9(9, 10)	0.50
Birth weight (g; n = 6465)	3480(3090, 3800)	3445(3118, 3799)	0.57
Multiple births			0.33
No	2243(96%)	3963(96%)	
Yes	89(4%)	179(4%)	
Any complications of labor/delivery	691(30%)	1200(29%)	0.58
Delivery method (n = 6426)			0.20
Vaginal	1907(82%)	3435(84%)	
C-section	409(18%)	675(16%)	
Anesthesia exposure			<.001
No exposures	2049(88%)	3480(84%)	
1 exposure	228(10%)	516(12%)	
Multiple exposures	55(2%)	146(4%)	

*Values are n (%) for categorical variables and median (Q1, Q3) for continuous variables. 315 subjects were not included in the analysis because enrollment status was not known at the time of sampling.

^†^P-values are from chi-square tests for categorical variables and Wilcoxon rank-sum tests for continuous or ordinal variables. When analyzing categorical variables, unknown or not specified categories are excluded from the analysis.

### Results of propensity matching

For those members of the birth cohort who were enrolled in ISD 535, 516 (12%) and 146 (4%) had single and multiple exposures to general anesthesia prior to age 3, respectively. Of the 50 possible strata defined by the combination of the propensity score quintiles, 15 (30%) strata were excluded because they did not contain at least one child from all three exposure groups. The strata excluded were: F(1,4), F(1,5), F(2,4), F(3,1), F(3,4), F(4,1), F (5,5), M(1,2), M(1,3), M(1,4), M(1,5), M(2,4), M(2,5), M(5,1) and M(5,5). The excluded strata included 50 (9.7%) and 20 (13.7%) of those with single and multiple exposures respectively. After propensity matching, the study cohort consisted of 126 children who had two or more general anesthesia exposures, 466 children who received general anesthesia only once, and 465 children unexposed to general anesthesia before age 3 (a total of 1,057 children).

Among the 1,057 children in the study cohort, baseline characteristics, including birth information, ADG comorbidity cluster variables, and HOUSES index were compared across the three exposure groups (Table 2). Characteristics including race and ethnicity, birth weight, estimated gestational age, mother’s age at delivery, and education levels for both parents did not differ among exposure groups. The frequencies of 28 ADG clusters were not different among the exposure groups for the total of 31 ADG clusters compared (S1 Table). Pregnancy is unlikely for the cohort children at baseline and therefore the ADG cluster for pregnancy was not compared. The HOUSES index, used as a measure of socioeconomic status, was not different across three exposure groups.

**Table 2:** Characteristics of the study cohort consisting of multiply exposed, singly exposed, and unexposed children (n = 1057).*

	No exposure (N = 465)	Single exposure (N = 466)	Multiple exposures (N = 126)	*P* Value†
Sex				§
Male	274(59%)	279(60%)	64(51%)	
Female	191(41%)	187(40%)	62(49%)	
Hispanic origin of father (n = 1011)				0.14
No	424(96%)	426(95%)	112(94%)	
Yes	11(2%)	10(2%)	0(0%)	
Not specified	8(2%)	13(3%)	7(6%)	
Hispanic origin of mother				0.33
No	449(97%)	455(98%)	125(99%)	
Yes	15(3%)	11(2%)	1(1%)	
Not specified	1(0%)	0(0%)	0(0%)	
Race of father, white (n = 1011)				0.11
No	30(7%)	25(6%)	14(12%)	
Yes	402(91%)	410(91%)	98(82%)	
Not specified	11(2%)	14(3%)	7(6%)	
Race of mother, white				0.09
No	40(9%)	25(5%)	14(11%)	
Yes	422(91%)	439(94%)	112(89%)	
Not specified	3(1%)	2(0%)	0(0%)	
Birth weight ≥ 2500g	409(88%)	422(91%)	110(87%)	0.30
Estimated gestational age (w)				0.45
<32	18(4%)	12(3%)	4(3%)	
32–36	55(12%)	51(11%)	13(10%)	
>36	392(84%)	403(86%)	109(87%)	
Mother's education (y)				0.98
<12	36(8%)	44(9%)	10(8%)	
12	80(17%)	83(18%)	24(19%)	
13–15	120(26%)	120(26%)	40(32%)	
16	132(28%)	123(26%)	29(23%)	
>16	89(19%)	88(19%)	21(17%)	
Unknown	8(2%)	8(2%)	2(2%)	
Father's education (y; n = 1011)				0.86
<12	14(3%)	17(4%)	8(7%)	
12	97(22%)	100(22%)	29(24%)	
13–15	101(23%)	105(23%)	25(21%)	
16	120(27%)	118(26%)	24(20%)	
>16	95(21%)	86(19%)	23(19%)	
Unknown	16(4%)	23(5%)	10(8%)	
Mother's age (y)	29.3(5.5)	29.2(5.7)	28.1(5.7)	0.56
HOUSES index (n = 951)	-0.0(3.2)	0.2(3.0)	-0.4(2.7)	0.81
Quartile of HOUSES index (n = 951)				0.11
1	98(23%)	103(24%)	35(32%)	
2	118(28%)	90(21%)	27(25%)	
3	105(25%)	105(25%)	25(23%)	
4	99(24%)	123(29%)	23(21%)	

* Values are n (%) for categorical variables and mean (SD) for continuous variables.

^†^P values are from Cochran-Mantel-Haenszel tests for categorical variables and generalized linear models for continuous variables taking into account the stratified design. Given the stratified design and variable number of children in each strata specific exposure group, p-values cannot be calculated directly from the data presented. When analyzing categorical variables, unknown or not specified categories are excluded from the analysis.

^§^Separate strata were created for males and females. Thus, each stratum was composed of a single sex.

### Comparison of children exposed to anesthesia who were and were not included in the study cohort

As noted above, 70 children who were enrolled in ISD 535 and exposed to general anesthesia were not included into the study cohort after propensity matching due to the lack of representation of all exposure groups in their sex-specific propensity strata, a potential source of bias. Comparing those included in the study cohort versus not, no differences were observed in birth and parental characteristics, including sex, parental races, estimated gestational age, parental education levels, mother’s age at delivery, and socioeconomic status (Table 3). The frequencies of 27 ADG clusters were not different among the exposure groups for the total of 31 ADG clusters compared (S2 Table).

**Table 3:** Characteristics of children exposed to general anesthesia who were and were not selected into the study cohort (n = 662).*

	Not selected into study cohort (N = 70)	Selected into study cohort (N = 592)	*P* Value†
Sex			0.05
Male	49(70%)	343(58%)	
Female	21(30%)	249(42%)	
Birth weight ≥ 2500g	58(83%)	532(90%)	0.07
Estimated gestational age (w; n = 660)			0.34
<32	3(4%)	16(3%)	
32–36	9(13%)	64(11%)	
>36	56(82%)	512(86%)	
Age of mother (y)	30(25, 34)	29(25, 33)	0.56
Hispanic origin of father (n = 634)			0.86
No	65(98%)	538(95%)	
Yes	1(2%)	10(2%)	
Not specified	0(0%)	20(4%)	
Hispanic origin of mother			0.73
No	69(99%)	580(98%)	
Yes	1(1%)	12(2%)	
Race of father, white (n = 634)			0.43
No	3(5%)	39(7%)	
Yes	63(95%)	508(89%)	
Not specified	0(0%)	21(4%)	
Race of mother, white			0.87
No	5(7%)	39(7%)	
Yes	65(93%)	551(93%)	
Not specified	0(0%)	2(0%)	
Mother's education (y)			0.53
<12	3(4%)	54(9%)	
12	13(19%)	107(18%)	
13–15	18(26%)	160(27%)	
16	20(29%)	152(26%)	
>16	12(17%)	109(18%)	
Unknown	4(6%)	10(2%)	
Father's education (y; n = 634)			0.57
<12	2(3%)	25(4%)	
12	17(26%)	129(23%)	
13–15	17(26%)	130(23%)	
16	15(23%)	142(25%)	
>16	11(17%)	109(19%)	
Unknown	4(6%)	33(6%)	
HOUSES index (n = 594)	-0.4(-1.9, 1.8)	-0.2(-2.0, 1.8)	0.84
Quartile of HOUSES index (n = 594)			0.84
1	15(24%)	138(26%)	
2	17(27%)	117(22%)	
3	14(22%)	130(24%)	
4	17(27%)	146(27%)	

* Values are n (%) for categorical variables and median (q1, q3) for continuous variables.

^†^ P values are from chi-square tests for categorical variables and Wilcoxon rank-sum tests for continuous or ordinal variables. When analyzing categorical variables, unknown or not specified categories are excluded from the analysis.

## Discussion

### Assessment of biases in study cohort selection

The population-based study design planned for this study has advantages in investigating the potential long-term neurodevelopmental outcomes of general anesthesia. In particular, it minimizes selection bias common to large pediatric referral centers. However, biases can still arise when the study sample of participants is not representative of the general population. Analyses of potential biases were facilitated by the fact that the birth cohort is fully characterized for every cohort member, including birth certificate information, perinatal medical records, and other important information such as address.

Approximately one-third (36%) of all children born in Olmsted County over the period of interest were not enrolled in ISD 535 during their fifth and sixth years. This may be explained by outmigration typical in any population. In addition, because healthcare is the major industry in the community, an additional proportion of Olmsted County residents of childbearing age are temporary personnel such as medical residents and contractors who are likely to move elsewhere after completion of their medical training or contractual work. In addition, although ISD 535 includes most of Olmsted County, there are portions of other public school districts also included in the county; because these school records were not available for review, these children were not included. Most available characteristics were similar in those children in the birth cohort who did and did not enroll. Although parents of those not enrolled were younger, the absolute differences in age were small. Children enrolled were also slightly more likely to be exposed to anesthesia prior to age 3 for reasons that are not clear.

### Propensity matching

An unavoidable limitation of observational studies in this area is that children who require anesthesia and surgery may be in some respects different from those who do not. This introduces the potential for confounding, as anesthesia exposure may simply be a marker for those children at risk for adverse outcomes. Propensity matching was utilized to select children so as to reduce the potential for confounding by identifying children at similar risk to receive anesthesia, based on a variety of characteristics. The lack of differences among exposure groups in the available characteristics suggests the success of propensity matching in these terms. However, an important limitation of propensity matching is that it may not be possible to find appropriate matches for children in the two “tails” of the propensity score distribution [21]. For our study, this limitation is complicated by the fact that we are balancing across 3 exposure groups. By creating strata based on the combination of two propensity scores we were able to identify a study cohort which was balanced across all groups. However, eliminating strata which did not have at least one individual from each exposure category resulted in the exclusion of 70 (10.6%) individuals exposed to general anesthesia. In addition, since the number of unexposed individuals was lower than that of exposed individuals for many strata our final study cohort did not include an equal number of exposed and unexposed individuals. The characteristics of the exposed children who were excluded were similar to those who were included (Table 3). We chose this as a conservative approach so that all strata would be informative for later comparisons of neurodevelopmental outcomes between those with single exposures and multiple exposures compared to no exposures. Nonetheless, the fact that some members of the cohort are excluded because similar members of all exposure groups could not be found represents a source of bias. In particular, it is possible that those exposed children who were excluded represent those at the greatest risk for adverse outcomes, potentially biasing against finding differences in outcomes between exposure groups.

### Limitations

In addition to the limitations of observational associative studies already discussed, there are other limitations inherent to the retrospective design of this study. Due to the retrospective nature of the study, misclassification of birth certificate and/or diagnostic coding could occur. For example, studies question the coding accuracy of this type of vital documents in some categories such as number of prenatal visits or birth injuries [22–24]. Similarly, the co-morbidity clusters utilized in this study rely mainly on the ICD-9 diagnostic codes, which are intended for billing purposes and could be inaccurate in a research context. In our study, however, the misclassification should occur in a non-systematic pattern independent of the school enrollment status and should not bias comparisons between ISD 535 enrollees and non-enrollees. In addition, although we have access to many baseline variables, some confounders that influence the likelihood of a child to enroll in ISD 535 remain unmeasured and unanalyzed and may affect future analysis of the association between anesthesia exposure and neurodevelopmental outcomes. With the population-based study design and access to integrated medical and school records, we believe many of these potential confounders can be accounted for in our later data analysis. As noted previously, we chose to eliminate anesthesia-exposed children who could not be propensity matched to at least one unexposed child from the cohort, which could introduce bias in later analysis, and the numbers of exposed and unexposed children were not equal in each stratum. Finally, although most characteristics of Olmsted County residents are similar to the rest of Minnesota and the upper Midwest, some differ from the US population as a whole [25].

## Conclusions

We describe the construction of a propensity-matched study cohort of 1,057 children born in Olmsted County, Minnesota with available school records who are singly-, multiply-, or un-exposed to general anesthesia prior to age 3, and analyze some of the potential biases associated with this construction. This study cohort will be used to determine the association between exposure to anesthetics used in contemporary practice and a variety of neurodevelopmental outcomes. Characterization of the constructed study cohort did not reveal significant biases that could compromise the planned analyses.

## Supporting Information

S1 Table Aggregated diagnosis groups (ADGs) of the study cohort consisting of multiply exposed, singly exposed, and unexposed children (n = 1057). (DOCX)

S2 Table Aggregated diagnosis groups (ADGs) of children exposed to general anesthesia who were and were not selected into the study cohort (n = 662). (DOCX)
