# Supplementary material for: Construction and Characterization of a Population-Based Cohort to Study the Association of Anesthesia Exposure with Neurodevelopmental Outcomes
Source: PLoS One. 2016 May 11;11(5):e0155288. doi: 10.1371/journal.pone.0155288 (PMC4864330; doi:10.1371/journal.pone.0155288)
Supplement: S1 Table — (DOCX) [file pone.0155288.s001.docx]

**S1 Table. Aggregated diagnosis groups (ADGs) of the study cohort consisting of multiply exposed, singly exposed, and unexposed children (n=1057)**

|  | No exposure (N=465) | | Single exposure (N=466) | | Multiple exposures (N=126) | | *P* Value† |
| --- | --- | --- | --- | --- | --- | --- | --- |
| Time Limited:Minor | 424 | (91%) | 430 | (92%) | 119 | (94%) | 0.55 |
| Time Limited:Minor-Primary Infections | 460 | (99%) | 463 | (99%) | 124 | (98%) | 0.80 |
| Time Limited:Major | 155 | (33%) | 159 | (34%) | 56 | (44%) | 0.68 |
| Time Limited:Major-Primary Infections | 172 | (37%) | 181 | (39%) | 67 | (53%) | 0.16 |
| Allergies | 106 | (23%) | 131 | (28%) | 38 | (30%) | 0.20 |
| Asthma | 64 | (14%) | 83 | (18%) | 32 | (25%) | 0.17 |
| Likely to Recur:Discrete | 235 | (51%) | 245 | (53%) | 75 | (60%) | 0.90 |
| Likely to Recur:Discrete_Infections | 443 | (95%) | 456 | (98%) | 125 | (99%) | 0.079 |
| Likely to Recur:Progressive | 0 | (0%) | 3 | (1%) | 0 | (0%) | 0.090 |
| Chronic Medical:Stable | 145 | (31%) | 134 | (29%) | 64 | (51%) | 0.21 |
| Chronic Medical:Unstable | 124 | (27%) | 123 | (26%) | 49 | (39%) | 0.89 |
| Chronic Specialty:Stable-Orthopedic | 15 | (3%) | 16 | (3%) | 11 | (9%) | 0.30 |
| Chronic Specialty:Stable-Ear,Nose,Throat | 127 | (27%) | 161 | (35%) | 63 | (50%) | 0.45 |
| Chronic Specialty:Stable-Eye | 142 | (31%) | 145 | (31%) | 54 | (43%) | 0.78 |
| Chronic Specialty:Unstable-Orthopedic | 1 | (0%) | 2 | (0%) | 0 | (0%) | 0.57 |
| Chronic Specialty:Unstable-Ear,Nose,Throat | 72 | (15%) | 120 | (26%) | 50 | (40%) | 0.19 |
| Chronic Specialty:Unstable-Eye | 37 | (8%) | 38 | (8%) | 19 | (15%) | 0.97 |
| Dermatologic | 150 | (32%) | 153 | (33%) | 48 | (38%) | 0.84 |
| Injuries/Adverse Effects:Minor | 245 | (53%) | 253 | (54%) | 73 | (58%) | 0.38 |
| Injuries/Adverse Effects:Major | 209 | (45%) | 233 | (50%) | 76 | (60%) | 0.32 |
| Psychosocial:Time Limited:Minor | 116 | (25%) | 123 | (26%) | 40 | (32%) | 0.99 |
| Psychosocial:Persistent/Recurrent,Stable | 100 | (22%) | 106 | (23%) | 40 | (32%) | 0.47 |
| Psychosocial:Persistent/Recurrent,Unstable | 11 | (2%) | 7 | (2%) | 7 | (6%) | 0.40 |
| Signs/Symptoms:Minor | 391 | (84%) | 405 | (87%) | 122 | (97%) | 0.096 |
| Signs/Symptoms:Uncertain | 431 | (93%) | 451 | (97%) | 126 | (100%) | 0.004 |
| Signs/Symptoms:Major | 308 | (66%) | 315 | (68%) | 102 | (81%) | 0.43 |
| Discretionary | 325 | (70%) | 365 | (78%) | 103 | (82%) | 0.004 |
| See and Reassure | 227 | (49%) | 231 | (50%) | 80 | (63%) | 0.66 |
| Prevention/Administrative | 464 | (100%) | 466 | (100%) | 126 | (100%) | 0.63 |
| Malignancy | 0 | (0%) | 2 | (0%) | 2 | (2%) | 0.27 |
| Dental | 5 | (1%) | 36 | (8%) | 12 | (10%) | <0.001 |

†P values are from Cochran-Mantel-Haenszel tests.
