# Supplementary material for: Construction and Characterization of a Population-Based Cohort to Study the Association of Anesthesia Exposure with Neurodevelopmental Outcomes
Source: PLoS One. 2016 May 11;11(5):e0155288. doi: 10.1371/journal.pone.0155288 (PMC4864330; doi:10.1371/journal.pone.0155288)
Supplement: S2 Table — (DOCX) [file pone.0155288.s002.docx]

**S2 Table. Aggregated diagnosis groups (ADGs) of children exposed to general anesthesia who were and were not selected into the study cohort (n=662)**

|  | Not selected into study cohort (N=70) | | Selected into study cohort (N=592) | | *P* Value† |
| --- | --- | --- | --- | --- | --- |
| Time Limited:Minor | 63 | (91%) | 549 | (93%) | 0.63 |
| Time Limited:Minor-Primary Infections | 69 | (100%) | 587 | (99%) | 1.00 |
| Time Limited:Major | 29 | (42%) | 215 | (36%) | 0.36 |
| Time Limited:Major-Primary Infections | 33 | (48%) | 248 | (42%) | 0.37 |
| Allergies | 20 | (29%) | 169 | (29%) | 1.00 |
| Asthma | 10 | (14%) | 115 | (19%) | 0.42 |
| Likely to Recur:Discrete | 37 | (54%) | 320 | (54%) | 1.00 |
| Likely to Recur:Discrete_Infections | 67 | (97%) | 581 | (98%) | 0.64 |
| Likely to Recur:Progressive | 9 | (13%) | 3 | (1%) | <0.001 |
| Chronic Medical:Stable | 28 | (41%) | 198 | (33%) | 0.28 |
| Chronic Medical:Unstable | 23 | (33%) | 172 | (29%) | 0.49 |
| Chronic Specialty:Stable-Orthopedic | 0 | (0%) | 27 | (5%) | 0.10 |
| Chronic Specialty:Stable-Ear,Nose,Throat | 23 | (33%) | 224 | (38%) | 0.51 |
| Chronic Specialty:Stable-Eye | 24 | (35%) | 199 | (34%) | 0.89 |
| Chronic Specialty:Unstable-Orthopedic | 0 | (0%) | 2 | (0%) | 1.00 |
| Chronic Specialty:Unstable-Ear,Nose,Throat | 21 | (30%) | 170 | (29%) | 0.78 |
| Chronic Specialty:Unstable-Eye | 12 | (17%) | 57 | (10%) | 0.059 |
| Dermatologic | 18 | (26%) | 201 | (34%) | 0.22 |
| Injuries/Adverse Effects:Minor | 42 | (61%) | 326 | (55%) | 0.37 |
| Injuries/Adverse Effects:Major | 40 | (58%) | 309 | (52%) | 0.38 |
| Psychosocial:Time Limited:Minor | 18 | (26%) | 163 | (28%) | 0.89 |
| Psychosocial:Persistent/Recurrent,Stable | 27 | (39%) | 146 | (25%) | 0.013 |
| Psychosocial:Persistent/Recurrent,Unstable | 3 | (4%) | 14 | (2%) | 0.41 |
| Signs/Symptoms:Minor | 65 | (94%) | 527 | (89%) | 0.22 |
| Signs/Symptoms:Uncertain | 66 | (96%) | 577 | (97%) | 0.42 |
| Signs/Symptoms:Major | 46 | (67%) | 417 | (70%) | 0.58 |
| Discretionary | 58 | (84%) | 468 | (79%) | 0.43 |
| See and Reassure | 37 | (54%) | 311 | (53%) | 0.90 |
| Prevention/Administrative | 69 | (100%) | 592 | (100%) | 1.00 |
| Malignancy | 3 | (4%) | 4 | (1%) | 0.028 |
| Dental | 12 | (17%) | 48 | (8%) | 0.024 |

†P values are from Fisher’s exact tests. One subject was missing all ADGs.
